# University Students’ Risk Perception, Protective Measures, and General Health During the COVID-19 Pandemic in Turkey

**DOI:** 10.1017/dmp.2022.216

**Published:** 2022-08-22

**Authors:** Sergul Duygulu, Emine Kuruca-Ozdemir, Yildiz Erdat, Deniz Kocoglu-Tanyer

**Affiliations:** 1 Faculty of Nursing, Department of Nursing Services Administration, Hacettepe University, Ankara, Turkey; 2 Faculty of Nursing, Department of Public Health Nursing, Selcuk University, Konya, Turkey

**Keywords:** university student, risk perception, protective measures, physical health, mental health, COVID-19, pandemic, cross-sectional

## Abstract

**Objective:**

This study aimed to investigate university students’ risk perception, protective measures, and general health during the coronavirus disease 2019 (COVID-19) pandemic in Turkey.

**Methods:**

The research sample consisted of 1920 university students. The data were collected through an online questionnaire.

**Results:**

A total of 56.6% of the students considered their risk of being infected with the COVID-19. The number of measures taken by students was lower than expected. Students’ increased anxiety perceived individual risk level, insufficient social support perceptions, and their perceptions of the current pandemic more serious than previous epidemics affect the number of measures they take. Students had sleep and study problems, and suicidal thoughts in the social isolation period. Sex, studying in medicine, anxiety related to COVID-19, feeling unconfident in coping with the pandemic, social support, were determined to be risk factors regarding general health, sleep and study problems, and suicidal thoughts.

**Conclusions:**

The results of the study showed that the measures taken by university students were insufficient and the precautions were affected by many factors. It was determined that their health was adversely affected by the pandemic. University administrations and decision-makers should consider the risk factors to improve the students’ experiences in such pandemics and emergencies.

After the first coronavirus case was detected in China in December 2019, other cases were identified in different regions of the world. This has rapidly become a global issue. The World Health Organization declared it a pandemic on March 11, 2020.^1^

As there was no proven treatment of the coronavirus disease 2019 (COVID-19), everybody could not provide access to the vaccine, infection transmitted very rapidly and easily from person to person and affected many people in a very short time. These issues forced countries to take some measures such as the closure of entry and exit to the country, implementation of lockdown in risky areas, closure of some workplaces, or limiting their services on national and international scales.^2^ Even if these measures were aimed at the whole population, the elderly people, children, and young people were the target age groups regarding the severity of the disease and their possible role in transmission. The main goal of these measures was to decrease the mobilization of individuals and their social contacts.

One of the first measures taken by countries was to suspend education in higher education institutions and to continue education distantly and by digital opportunities. According to the report of UNESCO, schools were closed in 189 countries and various restrictions were introduced on social life as of April 2020.^3^ This unanticipated crisis and the restrictions implemented have changed the lives of university students to a great extent.^4^

## University Students’ Risk Perception and Compliance With Protective Measures

According to data published by UNESCO, the number of university students affected by the COVID-19 in Turkey is above 7 million.^5^ It is expected that the university students who are a part of the population, being relatively more informed, have a higher level of knowledge, attitude, and perception regarding the pandemic, and become a model for people they live with.^6^ When considering that the pandemic can be prevented by the population’s compliance with protective measures, motivation, and cooperation, university students are the key individuals who can lead to changes in society.

During the pandemic, university students returned to their family homes and continued their education through online possibilities. As students spent more time in online environments, this caused their risk perceptions to vary in line with the information and news in the media.^4^ As a result of this, students faced many different discourses/expressions and showed behavioral differences toward protective measures. Young and Oppenheimer (2009) argued that exposure to different information sources is a very important factor generally influencing people’s attitudes and views regarding health.^7^ A study conducted in Turkey reported that students’ compliance with protective measures is parallel to the course of COVID-19 and their present level of knowledge about COVID-19.^8^ Some authors have attempted to explain the students’ compliance with protective measures through the use of theories. The theory of planned behavior (TPB) and protective motivation theory (PMT) have attracted particular attention.^9–11^ TPB is made up of 3 components: attitude, subjective norm, and perceived behavioral control. Fan et al. (2021)^9^ thought that these components may be important in students’ intention to be vaccinated. Huang et al. (2021),^10^ and Wang et al. (2021)^11^ claimed that, within the context of PMT, threat appraisal and coping appraisal are the factors that influence an individual’s protective behavior intention.

Various studies were carried out across the world to determine the risk perceptions of university students about COVID-19 and their compliance with protective measures. The conclusions mostly report that university students have a high level of risk perception.^12–14^ Ding et al. (2020) urged that the risk perception positively affected students and encouraged them regarding the implementation of protective measures.^13^ A study from Jordan indicated that students showed appropriate behaviors (hand hygiene, social distancing, etc.) for protection from COVID-19 and avoided taking the risk.^15^ Other studies in India and the Philippines stated that students found the measures to comply with social distance, washing hands, wearing a mask, and staying at home were very effective and important for protection.^12,16^

## COVID-19 and General Health

Fear, anxiety, and stress are normal reactions given to perceived or real dangers in case of uncertainty or an unknown situation.^17^ However, the extension of the pandemic duration continues to become a psychological pressure source for individuals.^18^ For this reason, national and international health authorities attach importance to policies for the protection of mental health. A systematic review reports relatively high anxiety, depression, post-traumatic stress disorder, psychological distress, and stress symptoms in the general population during COVID-19 pandemic.^19^ Similar results were reported in the university students. Due to COVID-19, university students have higher anxiety^20^ and depression scores^21^ than the general population.^22^ Students also have feelings of exhaustion, loneliness, nervousness, and anger due to the fear of COVID-19.^23^ Furthermore, a significant increase of somatization, obsessive-compulsive disorder, sensitivity, phobic anxiety, paranoid ideation, and general severity index scores^24^ and difficulties with sleep and concentration.^25^ The mental health risk factors of the students that increase anxiety and stress levels are feeling extreme fear, being in the graduation year, and living in severely affected regions.^22^ The other factors are media sourced information,^8^ delay in academic activities,^26^ lack of personal protective equipment,^27^ believing that the pandemic is a conspiracy,^28^ and concerns on economic impacts.^29^ Additionally, international university students were found to be at higher risk of psychological distress as compared with their local counterparts.^30,31^ Moreover, anxiety and perceived stress were found to be high among university students who were studying in medicine.^32^

Campus life provides opportunities for an active life and physical activity for university students. However, off-campus education and social isolation caused by COVID-19 have affected university students’ physical health by directly influencing their daily life routines.^33^ Students stated that the chronic stress during the pandemic resulted in many physical symptoms such as headache, migraine, insomnia, digestive problems, hormonal imbalances, sinusitis, anemia, and fatigue.^34^ Sleep problems are another problem caused by COVID-19. An increase in bedtime hours, sleep latency, and wake-up time as well as worsening in sleep quality and insomnia symptoms have been reported.^35^ In addition, daytime sleep duration has increased,^34^ and sleep quality has decreased in some students despite an increase in sleep duration.^36^

## Present Study

University students are more likely to be affected by the COVID-19 pandemic than other groups in social and educational respects, during social isolation. Therefore, this study aimed to investigate the compliance of university students with risk perceptions and protective measures and the factors affecting these. The study also aimed to determine physical and mental health perceptions, sleep and study problems, and suicidal thoughts of university students and examine the risk factors regarding these issues. According to data provided by UNESCO, the number of university students affected by COVID-19 in Turkey is more than 7 million.^5^ Considering the number of these students, it is clear to addressing the problems of those is crucial.

### Research Questions

This study attempted to answer the 2 research questions:

1. What are the protective measures taken by students and what are their determinants of the number of protective measures?
2. What are the risk factors related to COVID-19 for physical and mental health problems?

## Methods

### Participants

The research was carried out in a state university in the Ankara province of Turkey. The university performs educational activities in education, science, engineering, health, sports, and social sciences, fine arts, performing arts, and music, and declared to be a “Research University” by the decision of the Council of Higher Education. The sample was a subset of participants in the SATU Project, a descriptive study aiming to investigate the attitudes and behaviors of university students from different countries during the COVID-19 pandemic. The population of the research consisted of a total of 36,744 undergraduate students studying at this university. When examining the characteristics of the population, the majority of them were female (59%) and students at the faculty of literature (16.8%). No sampling method was used in the study, all actively studying students were included. Incomplete filling out of the questionnaire used in the research was determined as the exclusion criterion. A total of 1920 students participated in the study. All of the students who completed the questionnaire were undergraduate students, and most of them were female (71.5%) and medical students (18.2%). The adequacy of the sample size was evaluated for multiple regression analysis used to answer the research questions. For multiple regression analysis, 19 independent variables, 95% power, 0.05 alpha level, and f2 = 0.016 were assumed and the sample size was determined as 1913, and the sample size was considered sufficient.

### Procedures

To carry out the study, permission was obtained from the Ethical Committee of the University (Decision no: 35853172-755.02.06). The questionnaire and invitation letter were sent to the e-mail addresses of students by means of the Registrar’s Office by using Google Forms (online survey application), and consent was received before starting to fill out the questionnaire. The anonymity of all data was ensured. Reminder e-mails were sent to students by means of the Registrar’s Office after 7 d, 15 d, and 21 d when the first invitation was sent. Data were collected between August 1 and August 30, 2020 (in the fifth month following the first case in Turkey). A total of 1920 participants responded to the questionnaire. To prevent missing data, all questions in the questionnaire were required to be answered. If a question had been left blank, an automatic warning was sent by the survey application.

### Measures

In the research, a structured questionnaire with 48 questions was used to measure awareness and risk perception regarding the COVID-19 pandemic among university students. The questions were adapted from the risk perception of Effective Communication in Outbreak Management for Europe (ECOM) during the H1N1 pandemic in Hong Kong. The English version was developed by faculty members at the Department of Nursing of National Cheng Kung University in Taiwan. The questionnaire was translated from English into Turkish by 2 faculty members at the Faculty of Nursing of Hacettepe University, and 1 expert on English Philology. After completing the translation, the first Turkish version was created and submitted to an expert on Turkish Language and Literature to evaluate the conformity of Turkish grammatical structure. Last edits were made following the suggestions of the expert. The Turkish version was back-translated and was compared with the original one by an English philology expert. Finally, consent was received from the researchers who developed the questionnaire by e-mail.

The independent variables of the study are age, sex, study program, main field of study, social support resources, anxiety, individual risk perception, seriousness perception, resources owned, individual competence perception, concern, the feeling of trust, and communication restrictions. The number of measures, perceived physical health, mental health, feelings in social isolation, sleep, suicidal thought, and study problems are dependent variables of this study.

### Statistical Analysis

The data were analyzed by using IBM SPSS Statistics 24 program. The *P* < 0.05 value was accepted for the significance level. Number, percentage, mean, and standard deviation were used. The determinants of the number of measures taken by the students against COVID-19 were evaluated by hierarchical regression analysis. In this regard, 3 models were established. In the first model, sociodemographic variables; in the second model, perceptions against COVID disease; in the last model, perceptions toward resources were included in the model. Categorical variables were coded as 0 and 1. Before regression analysis, linearity, multicollinearity, normality, homoscedasticity, autocorrelation, variance inflation factor, and condition index estimations were evaluated.

The risk factors regarding students’ health outcomes were evaluated with logistic regression analysis (enter model). In this analysis data related to age was considered to be a continuous variable, other data were analyzed categorically.

## Results

The age average of students was 21.26 ± 3.5 y. A total of 16.8% of students stated that they did not receive sufficient support from their families during the COVID-19. This rate was found to be 20.1% for friends and 35.5% for instructors. The mean anxiety score of students’ regarding COVID-19 was found to be 5.4 ± 23, and the rate of those anxious about being infected the next day was 82.3%. A total of 16.8% of students stated that they did not receive sufficient support from their families during the COVID-19, 20.1% from friends, and 35.5% from faculty members. Whereas 56.6% of them considered the risk of students being infected with COVID-19 the same as other people, 11.9% of them evaluated this rate higher. A total of 54.3% of them found this pandemic more serious than severe acute respiratory syndrome (SARS), and 77.0% of them evaluated it more serious than seasonal flu. The rate of the students who were self-assured in fighting with the COVID-19 was 42.2%.

When the measures taken by students against COVID-19 were considered, it was observed that 78.3% of them developed behaviors for wearing a mask, and the rate was found to be 73.1% for keeping away from crowded environments and 65.3% for washing hands more frequently. House cleaning (34.0%), indoor ventilation (37.3%), and restricting the use of health services (4.2%) were found to be less taken are less taken measures. The mean number of the measures taken by the students was specified to be 4.7 ± 2.7 (Table 1). A total of 71.9% of the students found existing sources sufficient in obtaining personal protective equipment. This rate was 74.3% for information and informing related to COVID-19, while they found financial (38.9%) and medical sources (47.2%) less sufficient. The source that was found the least sufficient was the psychological support provided (23.1%) (Table 1).

**Table 1.** Students’ perception regarding the measures and resources they took due to COVID-19

	Yes		No	
**Measures taken**	**Number**	**%**	**Number**	**%**
Wearing a mask	1503	78.3	417	21.7
Compliance with social distancing	1404	73.1	516	26.9
Washing hands	1254	65.3	666	34.7
Indoor ventilation	717	37.3	1203	62.7
House cleaning	653	34.0	1267	66.0
Restricting communication with neighbor	653	34.0	1267	66.0
Restricting intrafamilial communication	291	15.2	1629	84.8
Restricting communication with friends	981	51.1	939	48.9
Restricting communication with classmates	1003	52.2	917	47.8
Obtaining information	550	28.8	1370	71.4
Not going to an existing doctor’s appointment	80	4.2	1840	95.8

	**Mean**	**±SD**	**(Min**-	**Max)**
**Number of measures taken**	**4.7±**	**2.7**	**0.00**	**11.0**
	**Sufficient**		**Insufficient**	
	**Number**	**%**	**Number**	**%**
Personal protection (mask-disinfectant)	1380	71.9	540	28.1
Information and informing	1427	74.3	493	25.7
Physical sources	747	38.9	1173	61.1
Medical sources	907	47.2	1013	52.8
Psychological support	443	23.1	1477	76.9

The determinants of the number of measures taken by students against COVID-19 were evaluated by hierarchical regression analysis. The determinants were explained over 3 models. In the first model, sociodemographic variables and variables related to the perception/source of social support were included. This model suggested that being female (β = 0.051) and not feeling the social support from friends sufficient (β = 0.105) increased the measures taken (F = 4.275; R^2^ = 0.11). In model 2, The variables of anxiety, risk, seriousness, and competence perception experienced due to the COVID-19 outbreak were added. In this model, the variables increasing the number of measures taken were as follows: not feeling the social support from friends sufficient (β = 0.063), increasing anxiety score (β = 0.371), high individual risk perception (β = 0.100), and perceiving COVID-19 as a more serious disease than SARS (β = 105) and seasonal flu (β = 0.090). These variables explained 21% of the number of measures taken (F = 36.473; R^2^ = 0.212). In Model 3, variables related to the sufficiency of some sources were added. However, it was seen that these variables did not have a significant contribution (Table 2).

**Table 2.** Determinants of the number of protective measures taken by students

	Model 1			Model 2			Model 3		
**Variables**	**β**	**t**	**p**	**β**	**t**	**p**	**β**	**t**	***P***
Age	0.040	1.753	0.080	0.007	0.327	0.744	0.004	.197	0.844
Sex	0.051	2.217	0.027	−0.016	−0.772	0.440	−.019	−.904	0.366
Study in medicine	−0.020	−0.858	0.391	0.015	0.742	0.458	0.021	1.023	0.307
Social support-family	−0.016	−0.663	0.507	−0.016	−0.770	0.441	-0.016	−.729	0.466
Social support-friend	0.105	4.279	0.000	0.063	2.841	0.005	0.063	2.819	0.005
Social support-instructor	−0.024	−1.016	0.310	−0.032	−1.468	0.142	−0.031	−1.427	0.154
Anxiety level				0.371	16.373	0.000	0.370	16.316	0.000
Equal individual risk perception				0.034	1.408	0.159	0.035	1.464	0.143
Higher individual risk perception				0.100	4.037	0.000	0.098	3.939	0.000
Equal seriousness perception with SARS				0.024	0.829	0.407	0.024	.834	0.404
Higher seriousness perception than SARS				0.105	3.561	0.000	0.105	3.563	0.000
Equal seriousness perception with seasonal flu				−0.012	−0.421	0.674	−0.013	−0.430	0.667
Higher seriousness perception with seasonal flu				0.090	3.015	0.003	0.091	3.042	0.002
Individual competence perception				−0.012	−0.557	0.577	−0.010	−0.453	0.650
Insufficient number of mask							−0.040	−1.719	0.086
Insufficient information and informing							0.000	0.016	0.988
Insufficient financial support							0.044	1.821	0.069
Medical insufficiency							0.022	0.860	0.390
Psychological insufficiency							0.020	−0.859	0.391
	**F**	***P***	**R**	**R** ^2^					
Model 1	4.275	<0.001	0.115	0.013					
Model 2	36.473	<0.001	0.460	0.212					
Model 3	27.309	<0.001	0.464	0.215					

The risk factors for perceived physical health during the pandemic were being students in medicine, not perceiving social support of friends and instructors (odds ratio [OR]: 1.437; 95% confidence interval [CI] [1.106 - 1.867]), increasing anxiety score for COVID-19 (OR: 1.089; 95% CI [1.023-1.159]) and feeling incompetent to cope with the pandemic (OR: 1.389; 95% CI [1.160-1.663]). The risk factors for perceived mental health during the pandemic were as follows: being female student (OR: 1.500; 95% CI [1.193-1.886]), not perceiving social support of friends (OR: 1.430; 95% CI [1.104-1.851]) family (OR: 3.342; 95% CI [2.393-4.127]), and instructors (OR: 1.482; 95% CI [1.199-1.831]), increasing anxiety score for COVID-19 (OR: 1.110; 95% CI [1.057-1.666]) and feeling incompetent to cope with the pandemic (OR: 1.214; 95% CI [1.050-1.403]). Increasing age showed a protective characteristic (OR: 0.956; 95% CI [0.927-0.986]) (Table 3).

**Table 3.** Risk factors for perceived physical and mental health

Risk factors	Perceived physical health			Perceived mental health		
	OR	(95% CI)	***P***	OR	95% CI)	***P***
Age	0.974	(0.939-1.010)	0.156	0.956	(0.927-0.986)	0.004
Sex	0.943	(0.709-1.254)	0.688	1.500	(1.193-1.886)	0.001
Study in medicine	1.398	(1.025-1.906)	0.034	1.042	(0.807-1.345)	0.754
Social support-family	1.358	(0.990-1.862)	0.057	3.142	(2.393-4.127)	0.000
Social support-friend	1.582	(1.172-2.136)	0.003	1.430	(1.104-1.851)	0.007
Social support-instructor	1.437	(1.106-1.867)	0.007	1.482	(1.199-1.831)	0.000
COVID-19 general concern	1.089	(1.023-1.159)	0.007	1.110	(1.057-1.166)	0.000
Being infected the next day	1.107	(0.755-1.624)	0.602	1.129	(0.847-1.505)	0.407
Individual coping with pandemic	1.389	(1.160-1.663)	0.000	1.214	(1.050-1.403)	0.009
Coping status of city being lived	0.987	(0.794-1.226)	0.904	1.121	(0.949-1.323)	0.179
Coping status of the city, where university is located	1.071	(0.877-1.307)	0.502	1.092	(0.937-1.272)	0.261
Communication-neighbor, immediate circle	0.887	(0.660-1.190)	0.423	1.038	(0.821-1.312)	0.757
Communication-family	1.095	(0.767-1.562)	0.618	1.154	(0.861-1.546)	0.339
Communication-friend	1.097	(0.828-1.454)	0.517	1.093	(0.875-1.366)	0.432

The risk factors for sleep problems during the pandemic were found to be insufficient social support of family (OR: 1.651; 95% CI [1.160-2.351]) and of friends (OR: 1.4991; 95% CI [1.076-2.087]), increasing anxiety score for COVID-19 (OR: 1.113; 95% CI [1.054-1.176]), and feeling incompetent to cope with the pandemic (OR: 1.275; 95% CI [1.075-1.511]). The risk factors for suicidal thought during the pandemic were as follows: insufficient social support of family (OR: 2.711; 95% CI [2.055-3.577]), of friends (OR: 1.432; 95% CI [1.080-1.899]), and instructor (OR: 1.562; 95% CI [1.225-1.993]) and having communication restriction with family (OR: 1.476; 95% CI [1.063-2.047]). The communication restriction with friends was concluded to have decreased suicidal thoughts (OR: 0.727; 95% CI [0.558-0.947]). The risk factors for study problems were insufficient social support of family (OR: 1.370; 95% CI [1.028-1.825]) and of instructor (OR: 1.861; 95% CI [1.495-2.316]), feeling incompetent to cope with the pandemic (OR: 1.254; 95% CI [1.082-1.454]), and communication restriction with friends (OR: 1.655; 95% CI [1.330-2.059]). Female students were found to be in a low-risk group for study problems (OR: 0.742; 95% CI [0.594-0.928]) (Table 4).

**Table 4.** Risk factors for sleep problem, suicidal thought, and study problems

	Sleep problem			Suicidal thought			Study problems		
**Risk factors**	**OR**	**(95% CI)**	***P***	**OR**	**(95% CI)**	***P***	**OR**	**(95% CI)**	***P***
Age	0.997	(0.966-1.028)	0.828	0.992	(0.959-1.027)	0.652	1.006	(0.978-1.035)	0.664
Sex	1.207	(0.948-1.537)	0.127	0.875	(0.675-1.133)	0.311	0.742	(0.594-0.928)	0.009
Study in medicine	0.915	(0.692-1.210)	0.534	0.728	(0.527-1.007)	0.055	1.085	(0.843-1.397)	0.528
Social support-family	1.651	(1.160-2.351)	0.005	2.711	(2.055-3.577)	0.000	1.370	(1.028-1.825)	0.032
Social support-friend	1.499	(1.076-2.087)	0.017	1.432	(1.080-1.899)	0.013	1.258	(0.955-1.658)	0.103
Social support-instructor	1.262	(0.986-1.617)	0.065	1.562	(1.225-1.993)	0.000	1.861	(1.495-2.316)	0.000
COVID-19 general concern	1.113	(1.054-1.176)	0.000	1.026	(0.969-1.086)	0.385	1.033	(0.985-1.085)	0.182
Being infected the next day	1.095	(0.820-1.462)	0.539	1.164	(0.828-1.636)	0.382	1.155	(0.884-1.508)	0.292
Individual coping with pandemic	1.275	(1.075-1.511)	0.005	1.165	(0.983-1.380)	0.079	1.254	(1.082-1.454)	0.003
Coping status of city being lived	1.043	(0.878-1.239)	0.631	1.105	(0.909-1.343)	0.316	1.148	(0.982-1.342)	0.083
Coping status of the city, where university is located	1.044	(0.888-1.227)	0.604	0.860	(0.721-1.026)	0.095	1.022	(0.883-1.182)	0.774
Communication-neighbor, immediate circle	0.868	(0.662-1.138)	0.307	1.141	(0.865-1.505)	0.350	1.249	(0.984-1.586)	0.068
Communication-family	1.066	(0.752-1.513)	0.718	1.476	(1.063-2.047)	0.020	1.330	(0.972-1.821)	0.075
Communication-friend	1.261	(0.982-1.619)	0.069	0.727	(0.558-0.947)	0.018	1.655	(1.330-2.059)	0.000

## Discussion

The COVID-19 pandemic still has a huge impact on study life, daily life, and the educational sector. University students are especially dealing with the serious uncertainty of their educational and social life. For this reason, it was aimed to determine the compliance of university students with protective measures and the factors affecting these measures.

### Perceived Risk and Preventive Measures

It has been identified that the compliance of university students with protective measures has not been sufficient to prevent the outbreak. In general, the number of measures taken by students is below average (Table 1). With the early stages of the COVID-19 outbreak, many organizations have taken actions for protective measures of the pandemic, have prepared, and published a lot of printed, visual, and audio materials. Special information has been provided to university students.^37^ Despite these efforts, it has been found that a group of students has complied with measures, but all the students have not followed these measures, and sufficient awareness and consciousness have not been achieved for taking multiple measures.

There are different conclusions on this subject in the literature. Elhadi et al. (2020) and Alves et al. (2020) reported that the number of measures taken by students has always been under the average.^14,38^ However, the studies conducted in India, Jordan, the Philippines, and China have indicated students’ compliance to be 80% and above.^12,15,16,39^

In this study, the number of measures taken by female students is more. Similar to this result, many previous studies have also reported that female students have shown a better attitude toward implementing the measures.^13,38–41^ Raising the awareness of students will affect their level to take measures. Yet, it should be remembered that it may be necessary to develop different strategies to reach male students. The fact that students do not believe the social support they receive from their friends is insufficient has increased the number of measures taken (Table 3). It is known that adolescents, including university students, display more risky behaviors when they are together with their peers than when they are alone.^42,43^ It may have had a positive effect that students have obtained suggestions mostly from media, television, official publications by keeping away from social circles due to restrictions. In such a case, university students have preferred to act less risky instead of being affected by their peers. Anxiety, risk, seriousness, and competence perception are also among the variables that have affected the number of measures taken by students (Table 3). This may be explained by Health Belief Model. According to this model, some components motivate an individual to take a measure and to protect his/her life. Some of these components are perceived sensitivity, perceived seriousness, and self-efficacy.^44^

### Physical and Mental Health With Associated Factors

This study has also examined the physical and mental health perceptions, sleep problems, suicidal thoughts, and study problems of university students in Turkey and determined the risk factors regarding these issues. Increasing age has shown a protective characteristic for mental health in this study. It is considered that not being able to start face-to-face education, get acquainted with university life, and meet the socialization needs due to the COVID-19 increased their stress levels and resulted in deterioration of mental health perception.

Being a female student is a risk factor for mental health perception. Prior studies on this subject have reported that women are at higher risk than men in terms of problems such as depression, loneliness, and daily life fatigue.^28,45^ Kuehner reports that women experience depression more often because of their greater exposure to stressors known to increase the risk of depression, such as violence, childhood sexual abuse, and gender inequality.^46^ On the other hand, other researchers have tried to explain this by fluctuations in women’s sex hormones, such as estrogen and progesterone, during their lifetime and more frequent exposure of women to gender-based violence than men.^47,48^ Female students have fewer study problems than male students in this study. In the related literature, studies are showing that female students show better performance at school.^49,50^ The result achieved in our study is of importance in presenting sex differences in the study problems experienced by students studying at the university in case of a pandemic.

Being a student in medicine is a risk factor for physical health. It is disputable whether medical students can be assigned to the care of COVID-19 patients due to the increasing number of cases and the need for physicians.^51^ Collado-Boira et al. have reported that final year nursing and medical students have tension, a sense of uncertainty, and fear regarding their immediate inclusion in the health system due to the pandemic.^52^ In addition, the news on the death of health-care professionals caused stress for these students.

Increasing anxiety score for the COVID-19 is a risk factor for perceived physical and mental health and sleep problems. Previous studies have also shown that stress and anxiety increased due to the COVID-19 have caused mental problems such as depression and sleep problems,^35^ as well as physical problems such as headache, digestive problems, and fatigue.^34^ Feeling incompetent to cope with the pandemic has been identified to be a risk factor for perceived physical and mental health, sleep, and study problems due to the reasons such as increased fear, anxiety, and stress. When examining the health-protective behaviors of individuals, those with self-confidence in coping with the pandemic show more protective behaviors.^53^ On the contrary, behaviors concerning protecting physical and mental health may decrease due to reasons such as decreased motivation, fear, and anxiety in the individuals feeling incompetent to cope with the disease.^54^

The perceived social support of family, friends, and instructors is an important risk factor affecting students’ physical and mental health, sleep, and study problems. The problems in the transition to online education may be a reason why students felt insufficient social support. The studies on adolescents and youth have shown that social support and feeling socially connected during the COVID-19 quarantine is a protective factor for poor mental health^55^ and insomnia symptoms.^56^ Together with perceived less social support, having communication restrictions with family was the risk factor for suicidal thoughts. Because the continuity of education came to the forefront at the beginning of the pandemic, especially in online education, the need for students to access learning resources (Internet, computer, smartphone, educator, etc.) may have brought along mental health problems. In the literature, the cases have been reported, who tried to commit suicide as they could not participate in lessons due to the lack of a television or smart phone at their family houses or family conflicts about online education.^57,58^

Another result achieved is that communication restriction with friends has unexpectedly reduced suicidal thoughts. This result can be explained by the fact that, when students reduce their communication with their friends, their frequency of talking about the pandemic, the fear of being infected, and negative situations that may occur in the study life decrease. We consider that such conversations may cause an increase in suicidal thoughts by raising the level of stress, hopelessness, and fear related to COVID-19 as reported in prior studies.^59,60^

There are some limitations of the study. The cross-sectional study design cannot provide empirical evidence in causal relationship, and the instruments used in the present study did not have robust evidence in their psychometric properties. Additionally, the participants were recruited using convenience sampling, and the representativeness of the sample was low. The participants completed the questionnaires by means of online self-report and could have social desirability bias and single rater bias.

## Conclusions

It is identified that the compliance of university students with protective measures has not been sufficient, and the changing life habits with the pandemic is the risk factor for the health of the students. Findings suggest that educational institutions should consider the risk factors affecting the university student’s general health and living problems, and develop measures and policies providing social and professional support.

## Supplementary material

For supplementary material accompanying this paper visit https://doi.org/10.1017/dmp.2022.216. click here to view supplementary material
