# Supplementary material for: University Students’ Risk Perception, Protective Measures, and General Health During the COVID-19 Pandemic in Turkey
Source: Disaster Med Public Health Prep. 2022 Aug 22:1–8. doi: 10.1017/dmp.2022.216 (PMC9530369; doi:10.1017/dmp.2022.216)
Supplement: Supplementary file 1 [file S1935789322002166sup001.docx]

**Suplementary Tables**

**Table 1** Perception of anxiety, risk, seriousness and competence due to the COVID-19 outbreak

| **Characteristics** | **n (%)** |
| --- | --- |
| **Anxiety to be infected with this disease in a week** |  |
| No/Little | 1485(77.3) |
| Much/Always | 435(22.7) |
| **Anxiety scale (now) *mean±sd*** | 5.4±2.3 |
| **Individual risk perception** |  |
| Never/ not likely | 605(31.5%) |
| Equal probability | 1086 (56.6) |
| likely/quite likely/absolutely | 229 (11.9) |
| **Seriousness perception (According to SARS)** |  |
| Less serious | 314(16.3%) |
| Same | 776(40.4%) |
| More serious | 1043 (54.3) |
| **According to seasonal flu** |  |
| Less serious | 188(0.9) |
| Same | 253(13,1) |
| More serious | 1479(77.0) |
| **Individual competence perception** |  |
| I am very sure/sure | 810(42.2) |
| More or less | 759(39.5) |
| Not sure | 351 (18.3) |

**Table 2** Students' information requests for COVID-19 *

|  | **n** | **%** |
| --- | --- | --- |
| How does school manage academic classes during COVID-19 outbreak? | 1098 | 57.2 |
| How does school manage the students in dormitory during COVID-19 outbreak? | 807 | 42.1 |
| How is COVID-19 treated? | 705 | 36.8 |
| How does school manage clinical practices during COVID-19 outbreak? | 622 | 32.4 |
| Your rate being infected with COVID-19 | 568 | 29.6 |
| The rate of seriousness of COVID-19 | 381 | 19.9 |
| What is the incubation period (time between infection and symptoms)? | 277 | 14.5 |
| What are the symptoms of COVID-19? | 231 | 12.1 |
| What can you do to avoid being infected with COVID-19? | 217 | 11.4 |
| How is COVID-19 transmitted? | 123 | 6.5 |
| I do not need any information | 110 | 5.8 |
| Other | 61 | 3.2 |

*More than one option was marked

**Table 3** Change in students' physical and mental health perceptions

|  | **Before Pandemic** | **In Social Isolation** | |  | **McNemar test and p value** |
| --- | --- | --- | --- | --- | --- |
|  |  | Negative | Positive | **Total** |  |
| **Physical health** | Negative | 146 | 156 | 302 (15.7%) | 0,778 |
|  | Positive | 173 | 1445 | 1628 (84.3%) | p=0,378 |
|  | **Total** | **319 (16.6%)** | **1601 (83.4%)** | **1920 (100.0%)** |  |
| **Mental health** | Negative | 406 | 174 | 580 (30.2%) | 63,520 |
|  | Positive | 359 | 981 | 1340 (69.8%) | P=0,000 |
|  | **Total** | **765(39.8%)** | **1155 (60.2%)** | **1920(100.0%)** |  |
